# Mechanochemical Synthesis of Molecular Chemoreceptors

**DOI:** 10.1021/acsomega.4c06566

**Published:** 2024-12-06

**Authors:** Jakub S. Cyniak, Artur Kasprzak

**Affiliations:** Faculty of Chemistry, Warsaw University of Technology, Noakowskiego Str. 3, 00-664 Warsaw, Poland

## Abstract

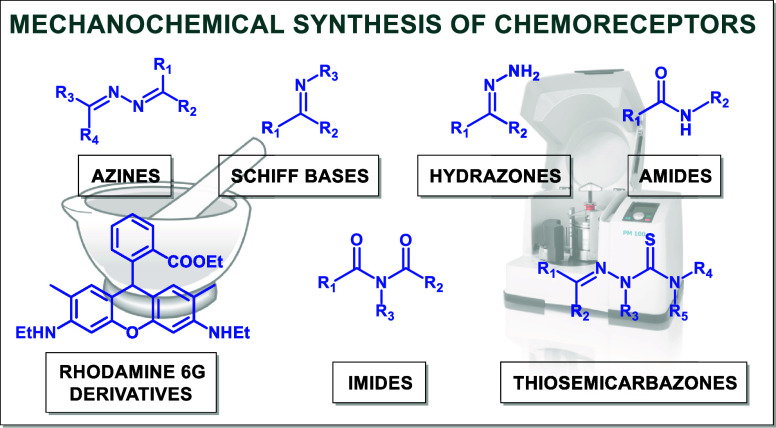

The design of environmentally friendly methods for synthesizing molecular receptors is an expanding area within applied organic chemistry. This work systematically summarizes advances in the mechanochemical synthesis of molecular chemoreceptors. It discusses key achievements related to the synthesis of chemoreceptors containing azine, Schiff base, thiosemicarbazone, hydrazone, rhodamine 6G, imide, or amide moieties. Additionally, it highlights the application potential of mechanochemically synthesized molecular chemoreceptors in the recognition of ions and small molecules, along with a discussion of the mechanisms of detection processes.

## 1 Introduction

Despite mechanochemistry being one of the first methods known to mankind for performing chemical reactions,^1^ this nonconventional synthesis technique is still recognized as a valuable synthetic tool and has been successfully used to develop new areas of chemistry by the next generation of scientists.^1−6^ Mechanochemistry stands out as a flagship method in green chemistry.^7^ In 2019, mechanochemistry was recognized by the International Union of Pure and Applied Chemistry (IUPAC) as one of the ten chemical innovations that are expected to change the world.^8,9^ Over the years, mechanochemistry has proven to be a particularly valuable synthetic tool. The many advantages of mechanochemical synthesis are particularly apparent when it is compared with synthesis in solution (the classical approach).^10−13^ The mechanochemical approach to organic synthesis fulfills many of the 12 postulates of Green Chemistry and allows for improved sustainability parameters.^14,15^ This is primarily due to a significant reduction in the use of (harmful) solvents or, in many cases, their complete elimination.^12,16−18^ Mechanochemistry also makes it possible in many cases to achieve increased yields by improving reaction selectivity^19−22^ and reducing the formation of byproducts, as well as provides access to molecules that could not be efficiently obtained, or even synthesized at all, using conventional solution-based approaches.^17,23^ Reduced synthesis times, compared to synthesis in solution, translate into reduced energy consumption, particularly when considering processes in which mechanochemistry has eliminated the need to carry out reactions at increased or reduced temperatures.^24^ For example, mechanochemistry has been successfully employed to synthesize molecules with a range of intriguing optical properties.^25−28^

In recent years, the design of molecular chemoreceptors has emerged as a rapidly growing area of research in organic and supramolecular chemistry.^29−31^ Molecular chemoreceptors are compounds capable of interacting with analytes through noncovalent forces. The feasibility of using organic molecular chemoreceptors for detecting various classes of analytes, particularly ionic species, has been demonstrated.^32−35^ Molecular chemoreceptors typically feature specific moieties, such as nitrogen- or oxygen-based structural motifs, that confer their recognition properties. Additionally, polyaromatic skeletons and other structural components may be included to fine-tune their properties, such as improving their photophysical properties. Binding phenomena in molecular chemoreceptors are commonly monitored spectroscopically (fluorescence *turn-off* or *turn-on* behaviors). Some chemoreceptors can also exhibit specific behaviors, such as the aggregation-induced emission (AIE) effect.^36−39^

The mechanochemical synthesis of organic molecular chemoreceptors represents an intriguing research area that combines the advantages of applying mechanochemical approaches in organic synthesis design with the utilization of such compounds in molecular recognition processes. The aim of this work is to systematically summarize advances in the field of the mechanochemical synthesis of molecular chemoreceptors. Literature reports, mostly from the past decade, were reviewed, grouping the synthesized chemoreceptors into seven general classes: azines, Schiff bases, thiosemicarbazones, hydrazones, rhodamine 6G derivatives, imides, and amides. The designed mechanochemical synthesis approaches are discussed, and their results are compared to outcomes from conventional solution synthesis. Additionally, the application of synthesized chemoreceptors as fluorescent probes, primarily for the recognition of ionic species, is discussed. The properties of chemoreceptors and the mechanism of their molecular recognition processes are also highlighted.

## 2 Mechanochemical Synthesis of Chemoreceptors

### 2.1 Azines

Azines, organic compounds containing a R^1^,R^2^C=N–N=CR^3^,R^4^ motif,^40^ have attracted the attention of researchers in recent years due to their interesting possibilities related to the synthesis of heterocyclic compounds or frameworks (COFs, MOFs), as well as prospective biological properties (synthesis of pharmaceuticals and other biologically active agents).^41^ Many examples of mechanochemical synthesis of azines as a general class of compounds have been reported,^42−46^ together with the examples of syntheses of functional materials, such as MOFs^47^ and Active Pharmaceutical Ingredients (APIs).^48^

**Figure 1:**
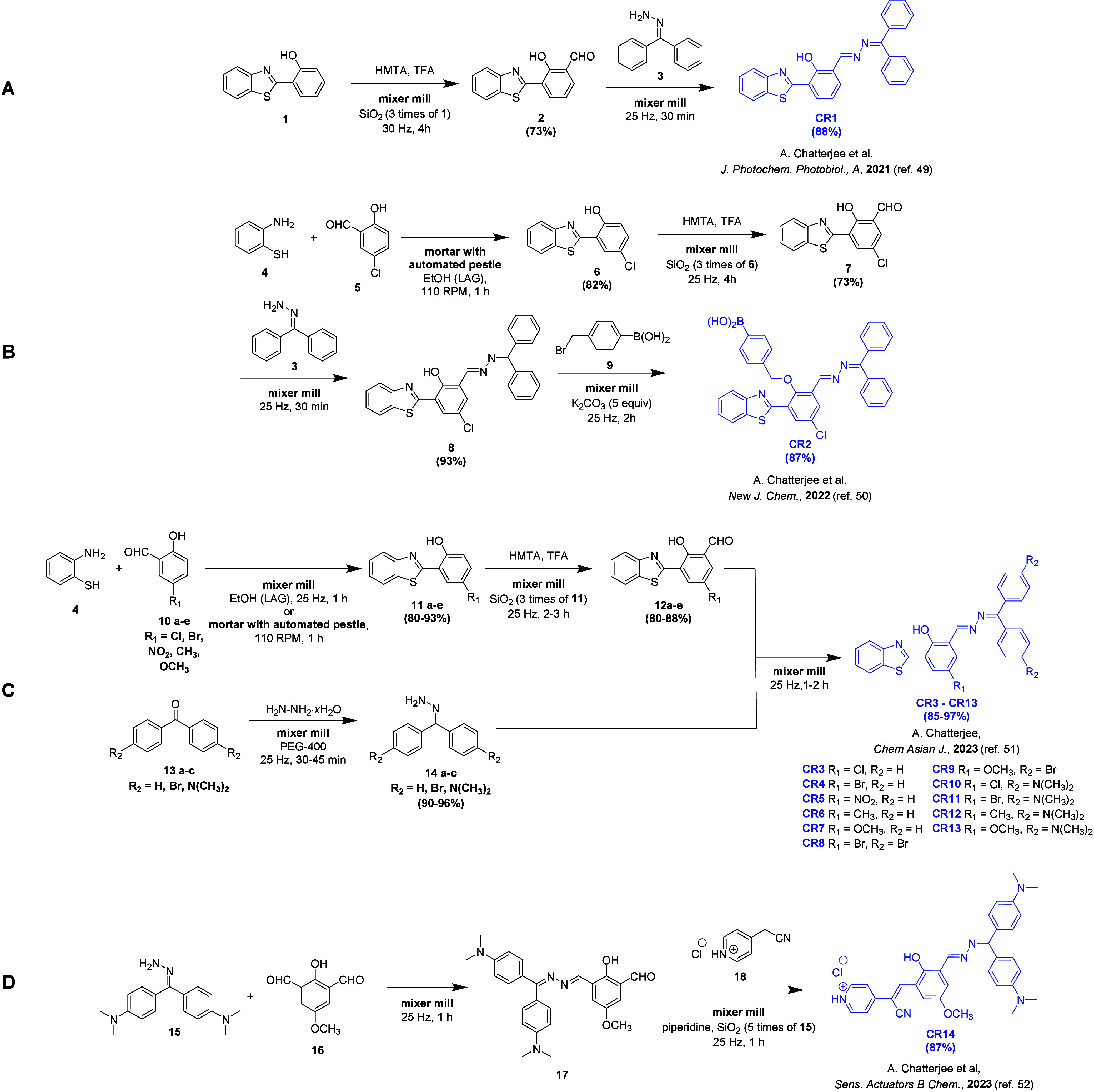
Synthetic pathway for receptors containing azine moiety: (A) **CR1**, (B) **CR2**, (C) **CR3–CR13**, and (D) **CR14**.

In a series of articles published between 2021 and 2023,^49−52^ Chatterjee and co-workers presented the mechanochemical synthesis of chemoreceptors containing an azine moiety (Figure 1A–D). Mechanochemical reactions were conducted using a mixer mill or in a mortar equipped with an automated pestle. Most of the presented chemoreceptors (**CR1–CR13**; Figure 1A–C) featured a hydroxyphenyl benzothiazole moiety.

For the synthesis of chemoreceptor **CR1** (Figure 1A), commercially available 2-(2-hydroxyphenyl)benzothiazole (**1**) was utilized. Subsequent chemoreceptors, **CR2**–**CR13** (Figure 1B–C), were synthesized using 2-(2-hydroxyphenyl)-benzothiazole derivatives (**6**, **11a**–**e**) prepared by grinding an equimolar mixture of aminothiophenol (**4**) with salicylaldehyde derivatives (**5**, **10a**–**e**) in the presence of small amounts of ethanol (liquid-assisted grinding, LAG). High yields of products (80–93%) were achieved. The resulting benzothiazoles (**6**, **11a**–**e**) were then converted into aldehydes using the Duff reaction. Developed in 1932,^53^ the Duff reaction selectively formylates the ortho position with respect to a strongly electron-donating group and has gained popularity in recent years in both classical approaches (reaction in solution)^54^ and mechanochemical synthesis.^55^ In these articles, benzothiazole derivatives (**1**, **6**, **11a**–**e**) were ground with hexamethylenetetramine (HMTA) in trifluoroacetic acid (TFA), where TFA served as a formylating agent and wetted the reaction mixture, aiding the grinding process (LAG approach). Silica gel (three times the weight of the starting material) was used as an additive to assist the grinding process. Such additives have been shown to improve, in some cases, the yield of mechanochemical reactions.^56^ The reactants were ground in a mixer mill for 2–4 h at 25–30 Hz, providing aldehydes with high yields (73–88%).

Benzophenone hydrazone derivatives (**14a**–**c**) were also prepared in a mechanochemical reaction by grinding benzophenone derivatives (**13a**–**c**) with hydrazine hydrate in the presence of poly(ethylene glycol) (PEG-400), yielding products with yields exceeding 90%. The synthesis of azines (chemoreceptors **CR1** and **CR3–CR13**, as well as starting materials for chemoreceptors **CR2** and **CR14**, compounds **8** and **17**, respectively) was carried out by grinding the reactants for 0.5–2 h, providing access to the target azine products in very high yields (88–97%). Noteworthy, the obtained compounds were isolated by recrystallization from an ethanol–water mixture, further enhancing the ecological and economical aspects of the presented method. Chemoreceptors **CR3**–**CR13** provided an intriguing example of the capabilities of mechanochemical synthesis, wherein the precursors used for chemoreceptor synthesis contained substituents of different electron characters. The presence of electron-withdrawing groups (Cl, Br, or NO_2_) and electron-donating groups (CH_3_, OCH_3_, or N(CH_3_)_2_) did not significantly influence the reaction yield. Chemoreceptor **CR2** was obtained in high yield (87%) by grinding 4-bromomethylphenylboronic acid (**9**) with the azine starting material (**8**) in the presence of potassium carbonate (K_2_CO_3_). Chemoreceptor **CR14** (Figure 1D) was synthesized in a two-step, one-pot reaction. First, the azine starting material (**17**) was synthesized, and then 4-(cyanomethyl)pyridinium chloride (**18**) was added to the same vessel and ground with piperidine and silica gel (five times the weight of **15**). Chemoreceptor **CR14** was isolated from the reaction mixture through simple extraction and solvent removal processes.

**Figure 2:**
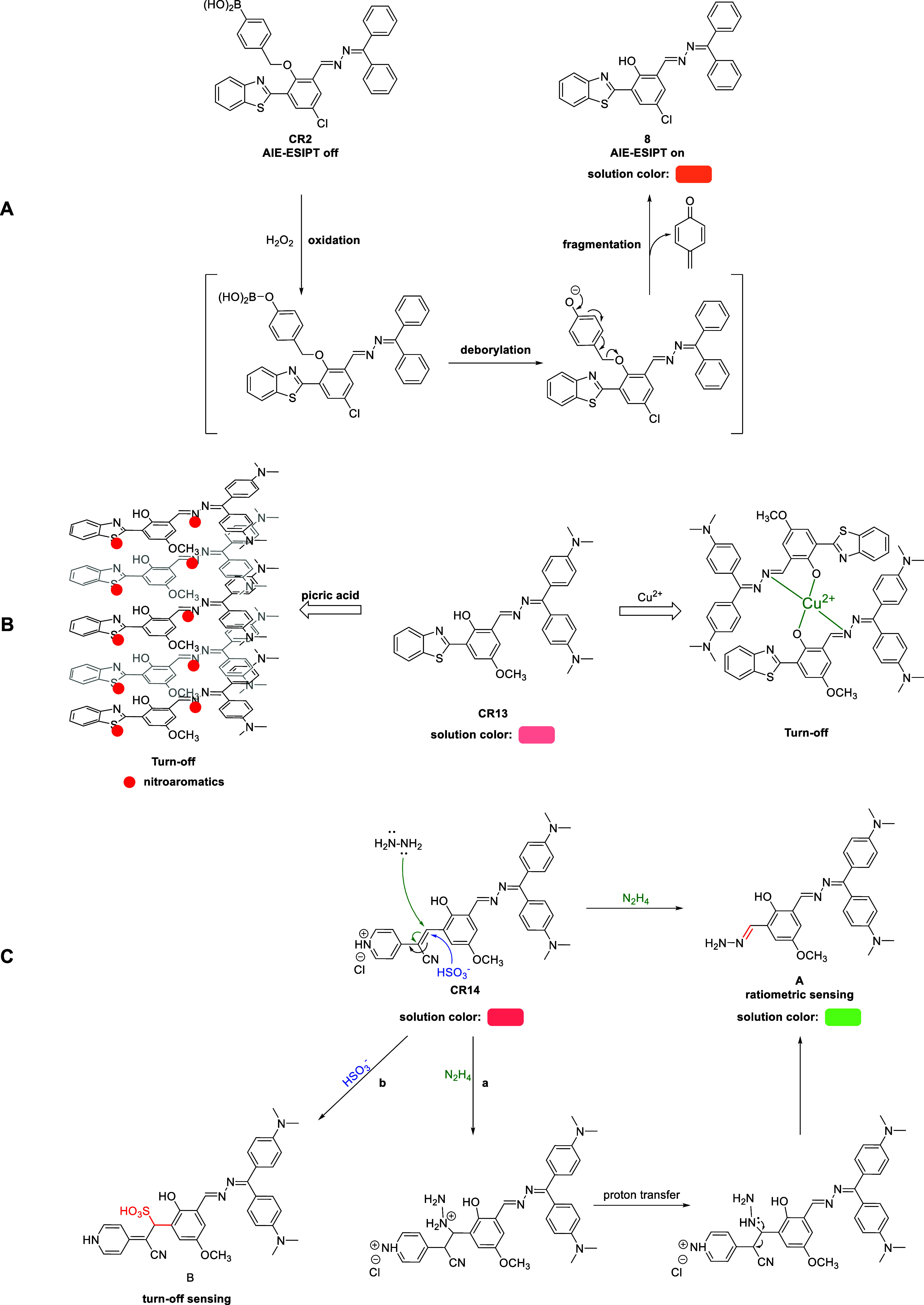
Proposed mechanisms of analyte detection by chemoreceptors: (A) **CR2**, (B) **CR13**, and (C) **CR14**.

It was demonstrated that chemoreceptors **CR1–CR14** could interact with various analytes through specific mechanisms of interaction. Chemoreceptors **CR1** and **CR3–CR13** selectively detected copper(II) cations (Cu^2+^) by chelating the Cu^2+^ cation through the phenolic hydroxyl (OH) group and the azine nitrogen atom. Two chemoreceptor molecules bound to one Cu^2+^ cation (Figure 2B). The Limit of Detection (LOD) value for **CR1** and **CR13** was determined to be 5.0 × 10^–9^ M and 0.6 × 10^–9^ M, respectively, whereas the binding constant of the formed complex (for **CR1**) was 3.37 × 10^–5^ M (the value of the binding constant for **CR13** was not determined). Noteworthy, due to its structural similarity to chemoreceptor **CR1**, compound **8** (the starting material of chemoreceptor **CR2**) can theoretically form complexes with Cu^2+^ cations. Similar behavior could also be expected for chemoreceptor **CR14**, but studies in this area have not been conducted. Chemoreceptor **CR2** was used to selectively detect hydrogen peroxide (H_2_O_2_) in aqueous samples. **CR2** did not emit light in organic solvents or aqueous–organic mixtures because the aggregation-induced excited-state intramolecular proton transfer (AIE-ESIPT) effect was not observed due to the lack of a phenolic hydroxy group. Detection of H_2_O_2_ was conducted in 1% DMSO in PBS (phosphate-buffered saline; pH = 7.4). H_2_O_2_ spontaneously cleaved the benzylboronic acid moiety, which restored **CR2** to fluorescent starting material **8** (Figure 2A). The LOD was 3.9 × 1 × 10^–8^ M.

Chemoreceptors **CR3–CR13** demonstrated a dual detection mechanism (Figure 2B). These chemoreceptors not only formed chelate complexes with Cu^2+^ but also detected nitroaromatic compounds (NACs). Electron-deficient NACs (e.g., 2,4,6-trinitrophenol) underwent noncovalent interactions with electron-rich chemoreceptors **CR3–CR13**, resulting in fluorescence quenching. Studies on chemoreceptor **CR13** (characterized by the highest emission intensity in the series) demonstrated its ability to selectively detect picric acid (2,4,6-trinitrophenol) in a DMF:H_2_O = 1:19 v/v system, with a LOD at the level of 10^–8^ M. The application of chemoreceptor **CR13** not only involved the use of classical techniques (spectrofluorimetry) but also allowed for the construction of a simple solid-phase analyte detection test for Cu^2+^. Silica-gel-coated TLC (thin-layer chromatography) plates were saturated with the chemoreceptor solution, and after drying, colorimetric detection of the presence and concentration (in the range of 1–50 ppm) of Cu^2+^ was possible.

**CR14** also exhibited selective recognition properties toward two different analytes, namely, hydrazine (N_2_H_4_) and the bisulfite anion (HSO_3_^–^) (Figure 2C). For the first analyte, the presence of hydrazine in the sample caused the spontaneous cleavage of the cyanopyridinium ethylene group, followed by the removal of the cyanopyridinium methylene unit, resulting in the formation of a hydrazone (Figure 2C, path a). The ratiometric response of the receptor was accompanied by a hypsochromic shift of emission from 718 nm (red) to 525 nm (green). On the other hand, the selective detection of the bisulfite anion was based on nucleophilic addition with the inclusion of the olefin bond (Figure 2C, path b). Notably, no conceptual interference between the analytes was observed due to the very low probability of the simultaneous presence of both oxidizing (HSO_3_^–^) and reducing (H_2_N_4_) agents in the same real sample.

### 2.2 Schiff Bases

**Figure 3:**
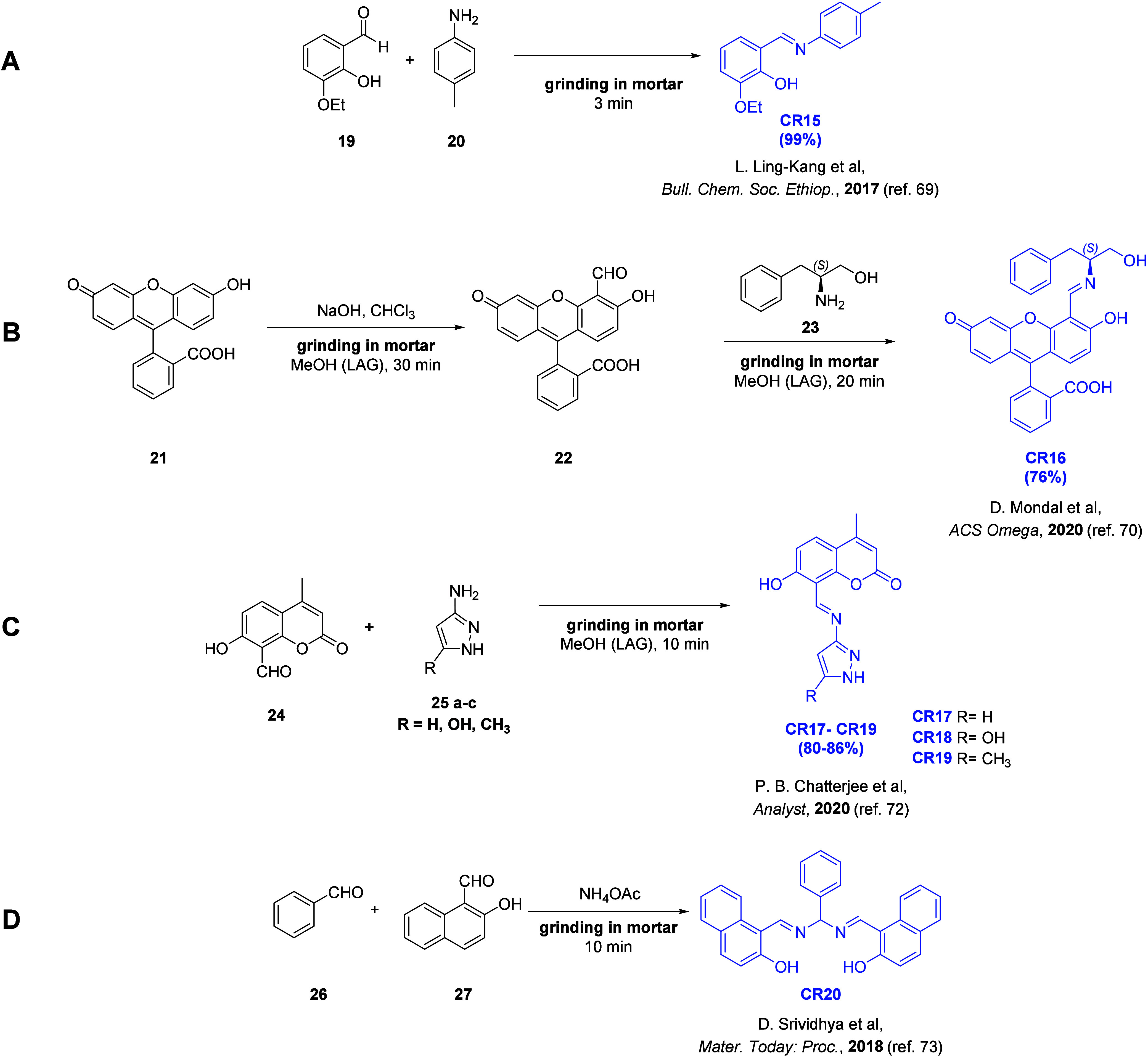
General structure of imine and synthetic pathway schemes of receptors containing Schiff base moiety: (A) **CR15**, (B) **CR16**, (C) **CR17–CR19**, and (D) **CR20**.

Schiff bases, with the general formula R^1^R^2^C=NR^3^ (where R^3^ ≠ H), are commonly synthesized using mechanochemistry.^57^ Not only simple derivatives containing the Schiff base motif^58^ but also polymers,^59^ porous materials,^60^ COFs,^61−63^ APIs,^64−66^ or luminescent materials are synthesized mechanochemically.^67,68^ Despite this, a limited, but still expanding, number of examples of the mechanochemical synthesis of chemoreceptors containing the Schiff base motif are known. Figure 3 depicts the synthesis schemes of six chemoreceptors (**CR15–CR20**) containing a Schiff base motif synthesized between 2017 and 2020.

The synthesis of chemoreceptor **CR15**(69) (Figure 3A) involved grinding 3-ethoxysalicylaldehyde with *p*-toluidine in a hand-held mortar. Grinding for just 3 min yielded **CR15** in excellent 99% yield. For the fluorescein-derived chemoreceptor **CR16** (Figure 3B), the Authors compared a solvent-based synthesis with a mechanochemical method.^70^ The first step was the synthesis of fluorescein monoaldehyde (**22**) by the Rimer–Tiemann reaction.^71^ The solution-based method involved refluxing fluorescein (**21**) for 12 h in chloroform in the presence of aqueous-methanolic sodium hydroxide (NaOH) solution with 15-crown-5 ether as a catalyst (28% yield). The mechanochemical version of this reaction provided a slightly higher yield (30%) while eliminating the need for solvents, high temperatures, and catalysts. It consisted of a 30 min grinding of fluorescein (**21**) with solid NaOH in the presence of a catalytic amount of acetic acid and a few drops of methanol (LAG approach). The solution-based synthesis of **CR16** involved stirring aldehyde (**22**) with l-phenylalanilol (**23**) in methanol for 12 h in the presence of a catalytic amount of diluted acetic acid (AcOH), yielding 68% yield. The mechanochemical synthesis involved grinding the starting material using a hand-held mortar for 20 min in the presence of a few drops of methanol (LAG approach), achieving a slightly higher yield (76%).

The synthesis of chemoreceptors **CR17**–**CR19** containing a pyrazole moiety (Figure 3C) was based on grinding of an aldehyde (**24**) with aminopyrazole derivatives (**25a**–**c**) in the presence of a few drops of methanol (LAG approach) for 10 min.^72^ The reaction products were purified by recrystallization from methanol, yielding **CR17**–**CR19** (80–86%).

Chemoreceptor **CR20** (Figure 3C) was synthesized by grinding benzaldehyde (**26**) and 2-hydroxynaphthaldehyde (**27**) in the presence of ammonium acetate.^73^ The starting materials were ground for 10 min, and then the viscous mixture was set aside at room temperature to solidify. **CR20** was purified by recrystallization from ethanol, but the reaction yield was not provided.

Chemoreceptors **CR15**, **CR16**, and **CR20** interacted with d-block metal cations by forming complexes with the inclusion of the nitrogen atom of the imine moiety and the phenolic hydroxyl (OH) group. In the case of chemoreceptor **CR16**, the hydroxyl group from l-phenylalaninol was also involved in the complexation phenomena.

Chemoreceptor **CR15** selectively formed complexes with copper(I) (Cu^+^) cations, accompanied by a color change of the receptor solution from being colorless to yellow. It was confirmed that the receptor was selective toward Cu^+^ and did not interact with other transition- and alkali metal cations. However, the Authors did not determine the thermodynamic parameters of the formed complexes.

Chemoreceptor **CR16** selectively complexed mercury(II) (Hg^2+^) cations with a stoichiometry of 1:1, resulting in a color change of the receptor solution from fluorescent green to pink. The interaction with Hg^2+^ was highly selective, as **CR16** did not interact with other d-block and f-block metal cations. The LOD value for this receptor was 3.4 × 10^–5^ M, and the binding constant was determined to be 1.7 × 10^–4^ M. The paper-strip sensor design and the possibility of removing Hg^2+^ ions from water using **CR16** were also presented.

Chemoreceptor **CR20** selectively forms complexes with copper(II) (Cu^2+^) and nickel(II) (Ni^2+^) cations, resulting in a color change of the receptor solution from colorless to violet and blue, respectively. The presence of other transition and alkaline metal cations as well as inorganic and organic anions in the sample did not induce a color change in the receptor solution, indicating no complexation phenomena for these interferents. The Authors determined that the **CR20** chemoreceptor forms 1:1 complexes with Cu^2+^ and Ni^2+^.

In contrast to other presented chemoreceptors containing a Schiff base moiety (Figure 3), chemoreceptors **CR17–CR19** do not interact with cationic species but rather with cyanide (CN^–^) anions. The postulated mechanism of interaction involves the deprotonation of the phenolic −OH group and the nucleophilic addition of CN^–^ to the methylylidene (=CH−) group from the imine moiety. It was also shown that the detection of the CN^–^ anion in human blood serum or the removal of CN^–^ from aqueous samples was possible using **CR17–CR19** chemoreceptors.

### 2.3 Thiosemicarbazones and Hydrazones

Thiosemicarbazones are organosulfur compounds, with the general formula H_2_NC(S)NHN=CR^1^,R^2^, that exhibit a variety of interesting properties such as the ability to complex transition metals^74−77^ or antimicrobial and anticancer properties^78−82^ but are not very commonly synthesized by the mechanochemical method.^83−85^ On the other hand, hydrazones with the general formula R^1^R^2^C=**NNH_2_** are more commonly synthesized using mechanochemical methods.^86^ There are known examples of APIs,^87,88^ MOFs,^89^ or metal complexes^90,91^ containing a hydrazone motif and synthesized mechanochemically.

**Figure 4:**
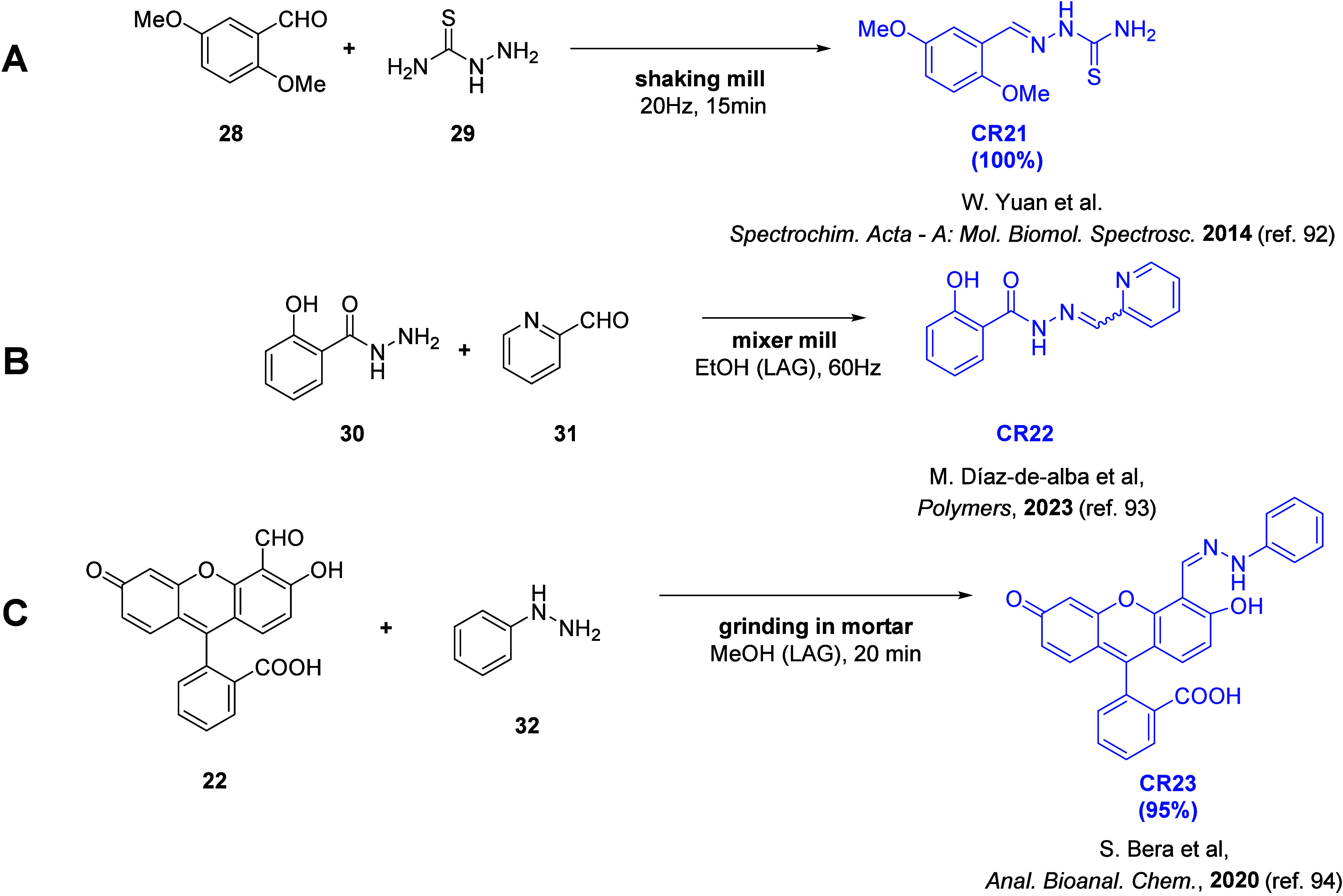
Synthetic pathway schemes of receptors containing a thiosemicarbazone moiety (A) **CR21** and hydrazone moieties (B) **CR22** and (C) **CR23**.

In 2014, Yuan and co-workers reported the mechanochemical synthesis of a chemoreceptor containing a thiosemicarbazone moiety, labeled as **CR21**(92) in Figure 4A. It was synthesized by solvent-free grinding of 2,5-dimethoxybenzaldehyde (**28**) with thiosemicarbazide (**29**) in a shaking mill (20 Hz). After 15 min, complete conversion of the starting materials was observed. Interestingly, an attempt to synthesize the chemoreceptor **CR21** in a planetary ball mill failed even when the reaction was conducted over 1 h or using the addition of solvent or an additive to assist the grinding. Chemoreceptor **CR21** selectively complexed Hg^2+^, with a stoichiometry of 2:1 (LOD = 1.85 × 10^–7^ M). The **CR21-Hg** complex was able to selectively interact with iodide (I^–^) ions, forming an insoluble salt (mercury(II) iodide, HgI_2_), which caused the breakdown of the **CR21-Hg** complex and a subsequent increase in the chemoreceptor’s fluorescence (*turn-on* behavior).

**CR22**(93) and **CR23**(94) (Figure 4B–C) containing a hydrazone moiety were obtained by grinding the aldehydes (**31**, **22**) with hydrazine derivatives (**30**, **32**). The **CR22** was obtained by grinding the starting materials in a mixer mill (60 Hz) in the presence of ethanol (LAG approach; reaction time, product purification method, and yield were not specified). For receptor **CR23**, grinding the starting materials in a hand-held mortar for 20 min with a couple of drops of methanol (LAG approach) provided a high (95%) reaction yield. The mechanochemical method was compared to the solution-based approach (86% yield achieved after 24 h). **CR22** was used to create an optical polymer inclusion membrane (PMI) sensor, which was used to detect iron(II) (Fe^2+^) ions. Like **CR16**, chemoreceptor **CR23** was able to selectively detect CN^–^ ions and was also shown to interact with fluoride (F^–^) ions. The formation of complexes with CN^–^ and F^–^ caused the change in the **CR23** solution color from colorless to pink and yellow, respectively. For both ions, the stoichiometry of the formed complexes was 1:3. The LOD values were determined to be at the level of 10^–5^ M for both ions, and the values of the binding constant were high, i.e., 1.32 × 10^13^ M^–3^ and 2.84 × 10^12^ M^–3^ for CN^–^ and F^–^, respectively. The feasibility of creating a paper-strip sensor and detecting CN^–^ in real samples (cigarette smoke extract) was also demonstrated.

### 2.4 Rhodamine 6G Derivatives

**Figure 5:**
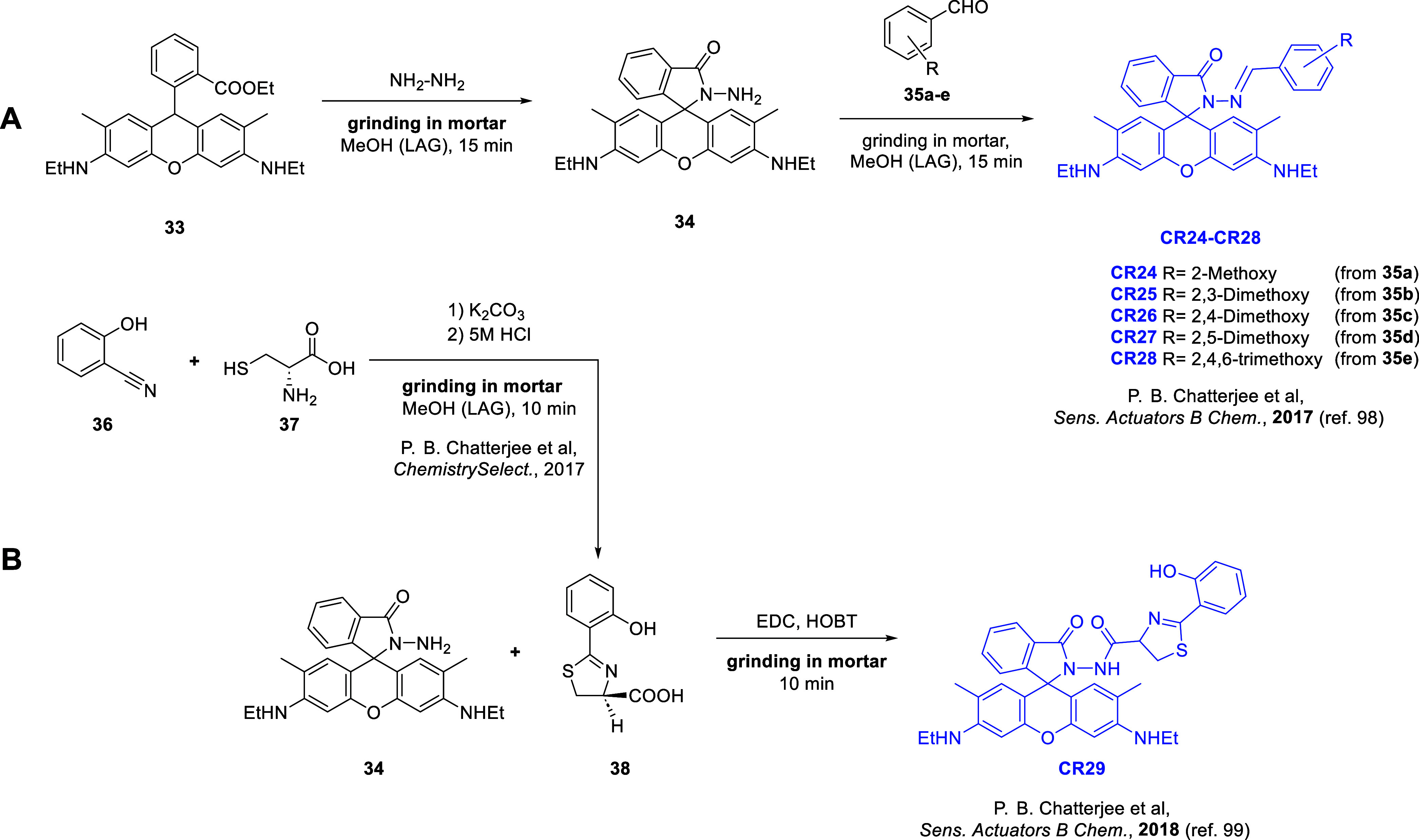
Synthetic pathway schemes of receptors containing the rhodamine 6G moiety: (A) **CR24**–**CR28** and (B) **CR29**.

Rhodamine 6G (Figure 5, structure **33**) belongs to the rhodamine group of dyes, which are triarylmethane derivatives of xanthene. Due to their unique emission properties, rhodamine derivatives are commonly used as fluorescent probes.^95−97^ The chemoreceptors outlined in this review have opened a new chapter in the mechanochemical modification of Rhodamine 6G.

In 2017, Chatterjee and co-workers presented the synthesis of a series of chemoreceptors (Figure 5A, **CR24–CR28**) containing a rhodamine 6G moiety.^98^ The authors presented, for the first time, a mechanochemical method for the synthesis of rhodamine 6G hydrazide (**34**), which involved grinding rhodamine 6G with hydrazine in a mortar in the presence of a few drops of methanol (LAG approach). The reaction product was purified by recrystallization from acetonitrile, achieving an excellent yield of 95%. The as-prepared hydrazide (**34**) was then subjected to a mechanochemical reaction with benzaldehyde derivatives (**35a**–**e**). The reactants were ground in a mortar with methanol (LAG approach) for 10–15 min. The mixture was then washed with cold methanol, dried, and recrystallized from acetonitrile. Chemoreceptors **CR24–CR28** were obtained in satisfactory yields (70–72%). Chemoreceptors **CR24–CR28** interacted selectively with copper(II) (Cu^2+^) cations, with only minor interference from other alkali and transition metal cations. Chemoreceptor **CR28** interacted selectively with Cu^2+^ without any interference. The mechanism of interaction of **CR24–CR28** with Cu^2+^ was investigated using mass spectrometry and infrared (IR) spectroscopy. It was concluded that the methoxy (−OCH_3_) group from the benzaldehyde derivative fragment, the carbonyl group, and the nitrogen atom from the hydrazide were involved in the formation of the complex. The binding constants and LOD values for the **CR24–CR28** chemoreceptors were determined to be at the level of 10^4^ M^–1^ and 10^–6^ M, respectively.

In 2018, Chatterjee and co-workers presented the synthesis of water-soluble chemoreceptor **CR29**.^99^ This molecule was obtained using the mechanochemical reaction of rhodamine hydrazide 6G (**34**) with aeruginic acid (**38**). First, 1-ethyl-3-(3-(dimethylamino)propyl) carbodiimide (EDC·HCl) was ground with triethylamine (TEA) in a hand-held mortar, followed by the addition of **38** and then the addition of hydroxybenzotriazole (HOBt), and further ground in the presence of a few drops of dichloromethane (CH_2_Cl_2_, LAG approach) for about 10 min. Hydrazide (**34**) was then added to the mortar and ground for an additional 10 min. The resulting pink powder was then extracted with CH_2_Cl_2_ and recrystallized from methanol to provide **CR29** in 68% yield.^100^

**Figure 6:**
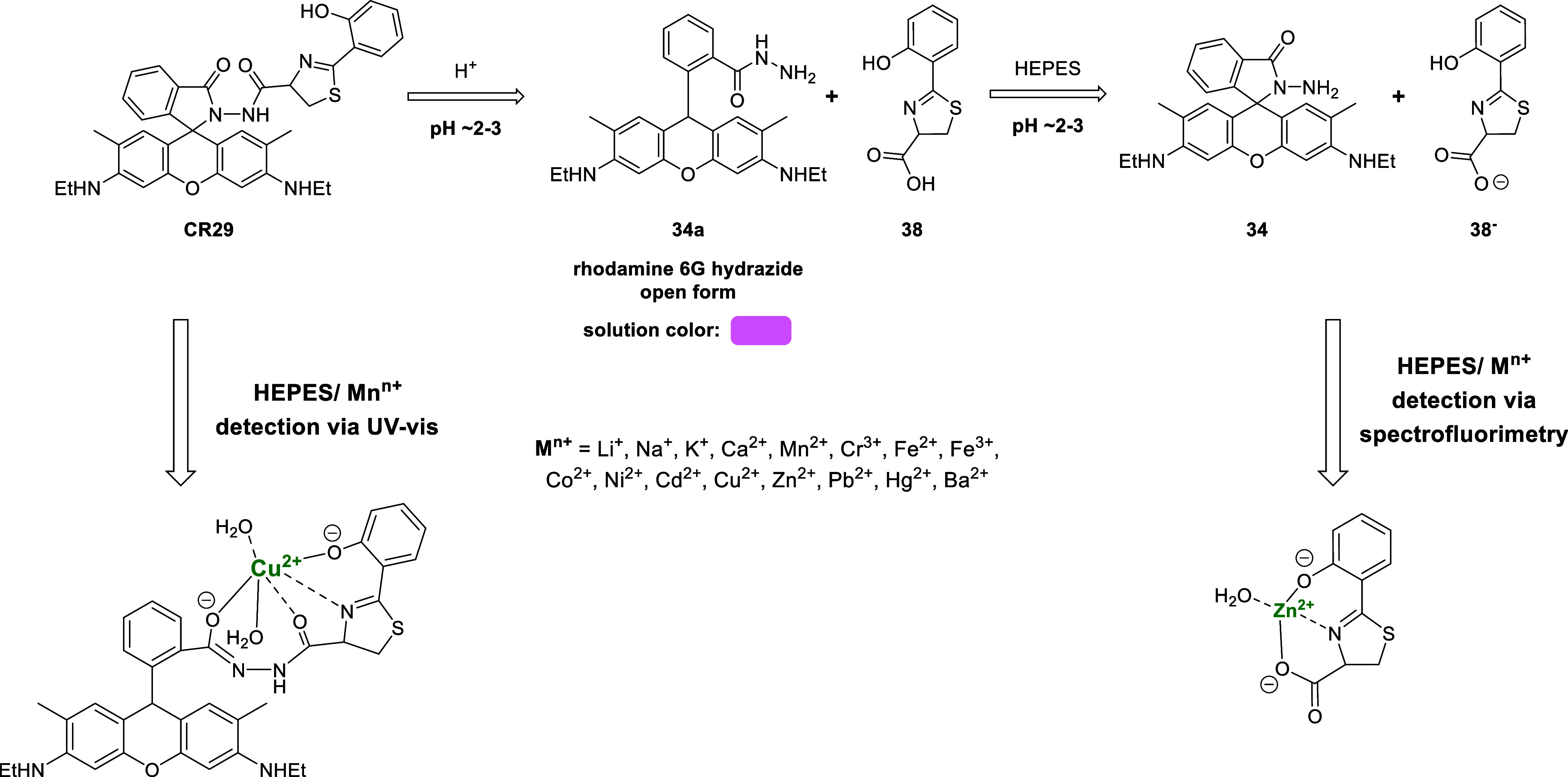
Proposed mechanisms of analyte detection by chemoreceptor **CR29**.

Chemoreceptor **CR29** exhibited an interesting dual model of interaction with cations (Figure 6). At pH 7.4 in 4-(2-hydroxyethyl)-1-piperazineethanesulfonic acid (HEPES) buffer, **CR29** selectively complexed Cu^2+^, and it was possible to detect this analyte using UV–vis spectroscopy. The binding constant and LOD were 0.6 × 10^–5^ M^–1^ and 3.7 × 10^–8^ M, respectively. On the other hand, if dilute inorganic acid was added to an aqueous solution (H_2_O, pH ∼ 2–3) of **CR29**, a color change of the solution from colorless to pink was observed. This behavior was determined (by mass spectrometry) to be related to the hydrolysis of the amide bond and the breakdown of **CR29** into dihydroaruginoic acid (**38**) and the open form of rhodamine hydrazide (Figure 6, **34a**). Restoring the pH to 7.4 caused the solution to become colorless. The hydrazide was converted to the spiro-lactam (closed) form, and the acid (**38**) was present in the anionic form. The released acid **38** could be used for the selective spectrofluorimetric detection of zinc(II) (Zn^2+^) cations.^101^

### 2.5 Imides and Amides

**Figure 7:**
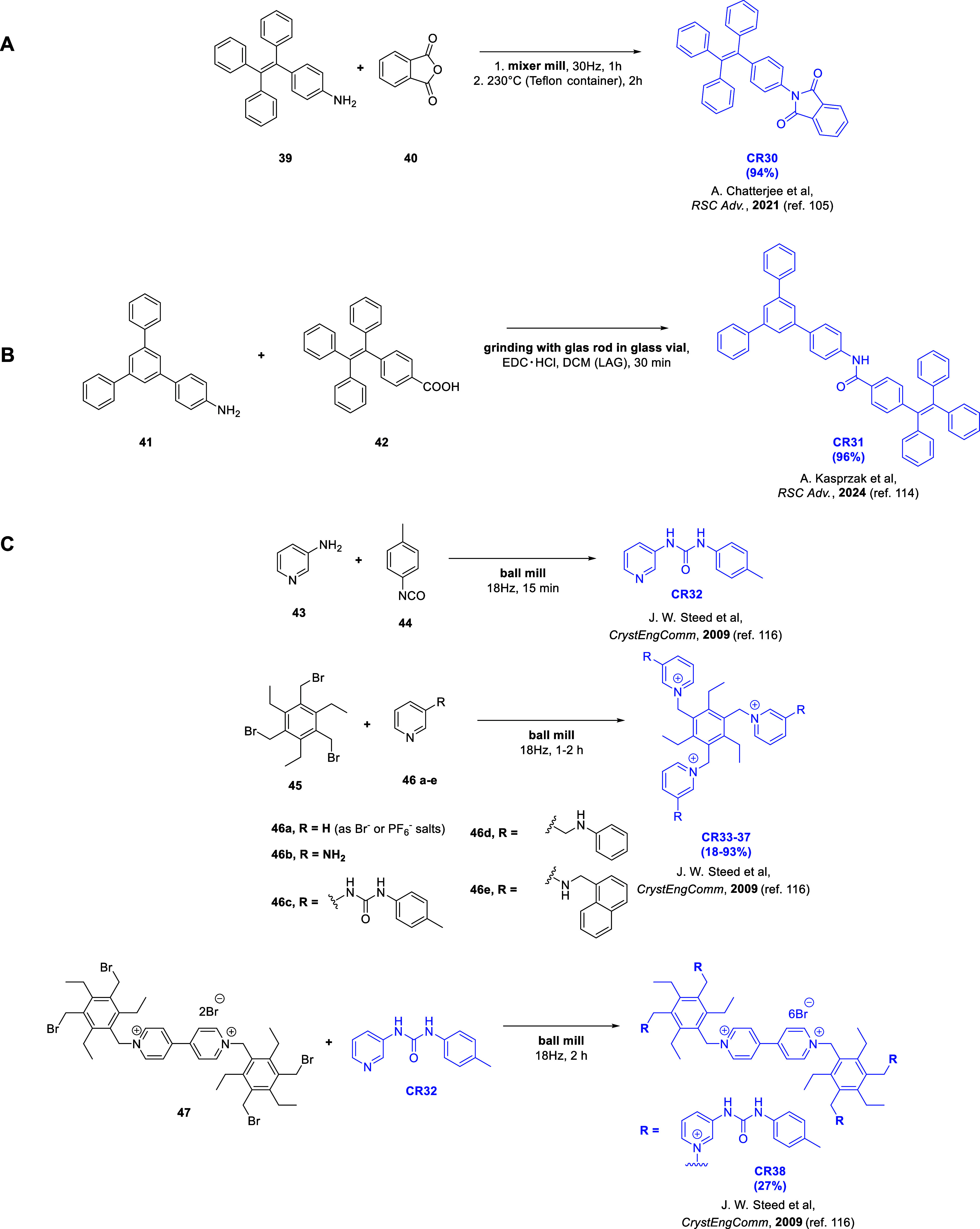
Synthetic pathway schemes of receptors containing an imide moiety (A) **CR30** and amide moiety (B) **CR31** or (C) **CR32–CR38**.

The mechanochemical synthesis of imides, with general formula (R^1^CO)**_2_**NR^2^, is very rarely mentioned in the literature and consists mostly of examples of the synthesis of dyes,^102^ APIs,^103^ polyimides,^104^ and chemoreceptors (Figure 7A).^105^ Examples of imide modification using a mechanochemical method were also reported.^106,107^ On the other hand, mechanochemical synthesis of amides (R^1^C(=O)NR^2^R^3^) has been extensively studied. In recent years numerous examples of mechanochemical syntheses of amide-based APIs,^108−110^ porous materials,^60^ and also the solid state synthesis of peptides^111−113^ were reported in the literature.

In 2021, Chatterjee and co-workers presented a mechanochemical synthesis of the chemoreceptor containing an imide moiety, **CR30** (Figure 7A).^105^ The synthesis involved grinding phthalic anhydride (**40**) with 4-(1,2,2-triphenylvinyl)benzamine (TPE–NH_2_; **39**) in a mixer mill for 1 h. The solid mixture was then transferred to a Teflon container and heated in a sand bath at 230 °C for 2 h. The resulting product was purified by recrystallization from an ethanol–water system. **CR30** was found to selectively detect hydrazine in aqueous samples (3% CH_3_CN in H_2_O or HEPES buffer). The interaction with hydrazine was not affected by the presence of various compounds in the sample, including amines, amino acids, transition and alkali metal ions, anions, or oxidizing agents. The postulated mechanism of hydrazine detection by **CR30** involved breaking the imide moiety with the release of AIE-active TPE–NH_2_. The first step in this process was the nucleophilic addition of hydrazine to the carbonyl carbon atom, followed by a nucleophilic attack on the carbonyl group with the release of phthalohydrazide and TPE–NH_2_. The authors also demonstrated the feasibility of using **CR30** to detect hydrazine in soil samples of living cells.

The synthesis of the AIE-active chemoreceptor **CR31** containing an amide moiety was presented in 2024 by Cyniak and Kasprzak (Figure 7B).^114^**CR31** was obtained by the direct mechanochemical reaction of an amine (**41**) and a carboxylic acid (**42**) in the presence of a coupling reagent (EDC·HCl) and a few drops of dichloromethane (LAG approach). The reactants were ground for 30 min with a glass rod in a glass vial to provide the main product in 96% yield. Interestingly, 30 min of grinding in a hand-held mortar provided the product with a lower yield (55%). Sonochemical synthesis and reactions in solution were also investigated. Mechanochemical synthesis was compared to solvent-based synthesis by calculating Green Chemistry parameters (Clark’s unified metrics toolkit).^115^ For this purpose, Atomic Economy (AE), Process Mass Intensity (PMI), and Reaction Mass Efficiency (RME) values were calculated, and the yields and reaction times of the syntheses were compared. For all analyzed parameters, mechanochemical synthesis delivered significantly better results as well as a better overall safety rating.

Taking advantage of the AIE activity of the chemoreceptor **CR31**, it was shown to interact (DMSO/H_2_O = 1/1 system, spectrofluorimetric studies) with a range of inorganic monovalent anions (Br^–^, I^–^, HSO_4_^–^, BF_4_^–^, H_2_PO_4_^–^, ClO_4_^–^, and CN^–^), as well as nucleotides, namely, AMP (adenosine monophosphate), ADP (adenosine diphosphate), NADP (nicotinamide adenine dinucleotide), FAD (flavin adenine dinucleotide), and ATP (adenosine triphosphate). The calculated binding constant values for both inorganic anions and nucleotides were around 10^–4^ M, with the highest values obtained for a perchlorate (ClO_4_^–^) anion and FAD, at 4.4 × 10^–4^ M and 8.8 × 10^–4^ M, respectively. The lowest LOD was observed for bromide (Br^–^) and FAD, at 0.17 × 10^–6^ M and 0.69 × 10^–6^ M, respectively. The contribution of the amide group to the interaction with the analyte was further confirmed by using ^1^H nuclear magnetic resonance (NMR) spectroscopy.

In 2009, Steed and co-workers described the synthesis of chemoreceptors containing a urea group, as well as trisubstituted and tetra-substituted chemoreceptors based on derivatives of pyridinium salts^116^ (Figure 7C). Reactions were conducted over 15 min to 2 h in a ball mill (18 Hz). The Authors did not conduct receptor studies using the obtained compounds but suggested the possibility of using them for anion detection.

## 3 Summary and Future Outlook

The state of the art in the mechanochemical synthesis of chemoreceptors is exclusively presented in this work. Highlighted reports, mostly from the past decade, clearly demonstrate the significant potential of mechanochemical synthesis in designing novel chemoreceptors with different analyte specificity or detection mechanisms. The capabilities of mechanochemistry related to the efficient synthesis of simple or structurally complex chemoreceptors using low-cost and easy-to-handle reaction systems, along with short reaction times and green process parameters, were emphasized. Numerous advantages of mechanochemical methods over classical solution methods are also demonstrated. It should be noted that the herein discussed area of research is attractive and still a growing field with many receptors yet to design. This manifests in the library of relatively simple derivatives; i.e., the mechanochemical syntheses summarized here are mostly devoted to the formation of carbon–nitrogen (C–N) bonds through well-established reactions. The chemoreceptors presented in this work were demonstrated to detect a specific number of analytes, consisting of mainly *d*-block metals, inorganic anions, small-molecule chemicals, or nucleotides (Table 1). It can be anticipated that the application of mechanochemical methods to create molecular chemoreceptors dedicated to recognizing environmentally important analytes will gain significant attention in the coming years.

**Table 1:** Summary of Chemoreceptors Presented in This Work: Synthesis Methods, Analytes, Thermodynamic Parameters

analyte	receptor	method of performing mechanochemical synthesis	binding constant	LOD	detection method	ref
Cu^2+^	**CR1**	mechanical milling	3.37 × 10^–5^ M	5.0 × 10^–9^ M	spectrofluorimetry	(49)
	**CR13**	mechanical milling	N/A	6 × 10^–10^ M	spectrofluorimetry	(51)
	**CR15**	hand-grinding in mortar	N/A	N/A	UV–vis spectroscopy	(69)
	**CR20**	mechanical milling	N/A	N/A	UV–vis spectroscopy	(73)
	**CR24 – CR28**	hand-grinding in mortar	0.88–1.54 × 10^3^ M	0.1–0.8 × 10^–6^ M	UV–vis spectroscopy	(98)
	**CR29**	hand-grinding in mortar	6.0 × 10^5^ M	3.70 × 10^–8^ M	UV–vis spectroscopy	(99)
Hg^2+^	**CR16**	hand-grinding in mortar	1.7 × 10^–4^ M	3.4 × 10^–5^ M	spectrofluorimetry	(70)
	**CR21**	mechanical milling	N/A	1.85 × 10^–7^ M	spectrofluorimetry	(92)
Ni^2+^	**CR20**	mechanical milling	N/A	N/A	UV–vis spectroscopy	(73)
Fe^2+^	**CR22**	mechanical milling	N/A	4.8 × 10^–5^ M	polymer inclusion membrane-based sensor	(93)
CN^–^	**CR17–19**	mechanical milling	2.19–5.20 × 10^2^ M	2.6 – 70 × 10^–9^ M	spectrofluorimetry	(72)
	**CR23**	hand-grinding in mortar	1.32 × 10^10^ M	1.25 × 10^–7^ M	spectrofluorimetry	(94)
HSO_4_^–^	**CR14**	mechanical milling	N/A	2.5 × 10^–8^ M	spectrofluorimetry	(52)
				2.4 × 10^–7^ M	UV–vis spectroscopy	
F^**–**^	**CR23**	hand-grinding in mortar	2.84 × 10^9^ M	5.9 × 10^–7^ M	spectrofluorimetry	(94)
N_2_H_2_	**CR14**	mechanical milling	N/A	4.0 × 10^–8^ M	spectrofluorimetry	(52)
				5.0 × 10^–7^ M	UV–vis spectroscopy	
	**CR30**	mechanical milling	N/A	6.4 × 10^–9^ M	spectrofluorimetry	(105)
H_2_O_2_	**CR2**	mortar with automated pestle, mechanical milling	N/A	3.9 × 10^–8^ M	spectrofluorimetry	(50)
picric acid (2,4,6-trinitrophenol)	**CR13**	mechanical milling	N/A	9.0 × 10^–8^ M	spectrofluorimetry	(51)
Br^–^, I^–^, BF_4_^–^, HSO_4_^–^, CN^–^, H_2_PO_4_^–^, ClO_4_^–^	**CR31**	hand-grinding with glass rod in glass vial	0.66–4.4 × 10^4^ M	0.16–19.9 × 10^–6^ M	spectrofluorimetry	(114)
nucleotides			0.76–4.4 × 10^4^ M	0.1–4.1 × 10^–6^ M		

## List of Key Abbreviations

LAG: Liquid Assisted Grinding
HMTA: Hexamethylenetetramine
TFA: Trifluoroacetic acid
LOD: Limit of Detection
AIE: Aggregation-Induced Emission
ESIPT: Excited-State Intramolecular Proton Transfer
NACs: Nitroaromatics Compounds
TEA: Triethylamine
HOBt: Hydroksybenzotriazole
EDC·HCl: 1-Ethyl-3-(3-dimethylaminopropyl)carbodiimide Hydrochloride
AcOH: Acetic Acid
API: Active Pharmaceutical Ingredient
COF: Covalent–Organic Framework
MOF: Metal–Organic Framework
